# Complete Chromatin Decondensation of Pig Sperm Is Required to Analyze Sperm DNA Breaks With the Comet Assay

**DOI:** 10.3389/fcell.2021.675973

**Published:** 2021-06-14

**Authors:** Jordi Ribas-Maynou, Ariadna Delgado-Bermúdez, Estela Garcia-Bonavila, Elisabeth Pinart, Marc Yeste, Sergi Bonet

**Affiliations:** ^1^Biotechnology of Animal and Human Reproduction (TechnoSperm), Institute of Food and Agricultural Technology, University of Girona, Girona, Spain; ^2^Unit of Cell Biology, Department of Biology, Faculty of Sciences, University of Girona, Girona, Spain

**Keywords:** sperm, DNA condensation, DNA damage, pigs, cattle, fertility

## Abstract

Sperm quality is usually evaluated prior to artificial insemination in farm animals. In addition to conventional semen analysis, other biomarkers, such as mitochondrial activity, integrity and lipid disorder of plasma membrane, generation of reactive oxygen species (ROS) and sperm DNA integrity, have been found to be related to fertility rates in different species. While mounting evidence indicates that the Comet assay is a sensitive method for the detection of DNA breaks, complete sperm chromatin decondensation is required in order to properly analyze the presence of single- and double-strand DNA breaks. In this sense, a previous study showed that longer lysis treatment with proteinase K is needed to achieve complete chromatin decondensation. The current work sought to determine which specific lysis treatment leads to complete chromatin decondensation in pig sperm, as this is needed for the measurement of DNA damage in this species. With this purpose, incubation with a lysis solution containing proteinase K for 0, 30, and 180 min was added to the conventional protocol. The impact of the DNA damage induced by hydrogen peroxide (H_2_O_2_; 0.01 and 0.1%) and DNAse I (1U and 4U) was also evaluated. Complete chromatin decondensation was only achieved when a long additional lysis treatment (180 min) was included. Furthermore, olive tail moment (OTM) and percentage of tail DNA (TD) indicated that a higher amount of DNA breaks was detected when hydrogen peroxide and DNAse I treatments were applied (*P* < 0.05). The comparison of treated and control sperm allowed defining the thresholds for OTM; these thresholds revealed that the percentage of sperm with fragmented DNA determined by the alkaline Comet does not depend on chromatin decondensation (*P* > 0.05). In conclusion, complete chromatin decondensation prior to alkaline and neutral Comet assays is needed to analyze DNA breaks in pig sperm.

## Introduction

Sperm account for the transport of paternal genes to the oocyte through the female tract. The challenge of producing a viable embryo is known to rely on the quality of both the oocyte and the spermatozoon. Assisted reproductive techniques (ART) allow overcoming some natural barriers of the sperm track toward the oocyte. In humans, ART are reserved to single women or infertile couples, who may be submitted to artificial insemination or may require complex and expensive procedures, such as *in vitro* fertilization (IVF) or intracytoplasmic sperm injection (ICSI; Bhattacharya et al., 2001; Palermo et al., 2017). Conversely, farm animals, such as pigs and cattle, are not bred through natural mating, but rather artificial insemination using liquid-stored or cryopreserved semen is conducted (Yeste et al., 2017; Schulze et al., 2019; Waberski et al., 2019). In this context, the semen production industry much relies upon two key pillars aimed at ensuring sperm quality: first, the genetic selection of individuals with the highest sperm quality and, second, the development of semen extenders and cryopreservation media that allow gamete preservation until insemination (Waberski et al., 2011; Zak et al., 2017).

Nowadays, sperm quality is routinely evaluated on the basis of their concentration, motility and morphology, usually with the assistance of a computer system/software (CASA) (Boe-Hansen and Satake, 2019; van der Horst, 2020). Also, a number of more complex tests requiring advanced equipment, including a flow cytometer or an epifluorescence microscope, have been developed in the last decades, with the aim of providing a wider picture of sperm function and fertilizing ability. These tests include the analysis of sperm viability, acrosome integrity, mitochondrial integrity and activity, and intracellular levels of reactive oxygen species (ROS), amongst others (Gillan et al., 2005; Boe-Hansen and Satake, 2019). While these variables are related to sperm fertilizing ability (Pérez-Llano et al., 2010), none is intended to assess DNA integrity, which is a key aspect for genetic quality and is related to embryo genetic alterations leading to reproductive failure (Lewis and Aitken, 2005; Simon et al., 2013; Casanovas et al., 2019).

Previous studies reported that viable spermatozoa can contain DNA breaks, supporting that apparently intact sperm with proper motility may bear fragmented DNA (Pérez-Llano et al., 2010; Samplaski et al., 2015; Muratori et al., 2020). DNA damage can occur in response to oxidative stress, but also as a result of enzymatic activity during chromatin remodeling and/or apoptotic-like processes (Aitken and De Iuliis, 2010; Sakkas and Alvarez, 2010; Bui et al., 2018; Ribas-Maynou and Benet, 2019). In humans, analysis of this genetic damage has been demonstrated to be a reliable biomarker for male fertility (Sharma et al., 2010; Simon et al., 2017b). Mounting evidence supports that distinct types of DNA breaks may be on the basis of different medical conditions, the single-strand breaks being associated to the lack of pregnancy and the double-strand ones being linked to lower embryo quality and miscarriage (Robinson et al., 2012; Borges et al., 2019; Ribas-Maynou and Benet, 2019). In production animals, such as pigs, sperm DNA fragmentation has been found to negatively affect the number of piglets born (Boe-Hansen et al., 2008; Myromslien et al., 2019); furthermore, a recent systematic review reported that DNA damage is a detrimental factor for IVF/ICSI outcomes in different mammals (Ribas-Maynou et al., 2020, 2021b).

Due to the replacement of most histones by protamines, sperm cells have a unique chromatin condensation that includes periodic toroidal structures of 50 kb in length (Barratt et al., 2010; Ward, 2010). This higher grade of chromatin condensation compared to somatic cells leads to an almost crystalline nucleus with a completely abolished transcriptional activity, and confers sperm DNA with a higher resilience to damaging agents (Enciso et al., 2011). Nevertheless, this high condensation represents a challenge for the study of sperm DNA, as complete chromatin decondensation is required to enable the access of DNA probes (Fernández et al., 2005; Mitchell et al., 2011; Simon and Carrell, 2013). Related to this, some authors proposed, through the “iceberg tip hypothesis,” that DNA condensation decreases the sensitivity of the detection of DNA breaks (Gosálvez et al., 2013; Cho et al., 2017). In effect, for some tests, such as sperm chromatin dispersion (SCD) or Comet, complete chromatin decondensation is needed to observe the presence/absence of haloes and the Comet tail, respectively (Enciso et al., 2006, 2011). In humans, the SCD test reveals that sperm with fragmented DNA do not exhibit haloes, whereas those with non-fragmented DNA do (Fernández et al., 2005). On the contrary, the use of the SCD test in pig sperm shows the opposite pattern: sperm with non-fragmented DNA are those without halo, and those exhibiting a halo are those that have their DNA fragmented (Pérez-Llano et al., 2010). Under the same decondensation treatment, these authors found very low sperm DNA fragmentation following the two-dimensional Comet assay (Pérez-Llano et al., 2010). While this low percentage of sperm with fragmented DNA was also observed in pigs by other authors (Hernández et al., 2006; Yeste et al., 2013a,b), a recent study by our research group has brought some light into the matter showing that pig sperm require longer lysis to completely decondense their chromatin, which is needed to evaluate DNA damage properly (Ribas-Maynou et al., 2021a). Interestingly, other studies conducted in pigs performed a longer lysis step, up to 4 h, and found higher values of sperm DNA fragmentation determined by the neutral Comet (Fraser and Strzezek, 2007a,b). Thus, evidence points out to insufficient chromatin decondensation as an explanation for the low percentages of sperm with damaged DNA determined thus far. For this reason, setting up a method that increases the sensitivity of DNA damage assessment is much needed for proper determination of sperm DNA integrity in this species. Also, because the alkaline Comet, rather than the neutral one, is the variant associated to human infertility (Ribas-Maynou et al., 2013), and previous studies in pigs used either the neutral Comet assay or the two-dimensional Comet assay, this work sought to improve the analysis of DNA fragmentation in pig sperm through the alkaline Comet. With this purpose, we evaluated how an extended lysis step with proteinase K affected the ability of the Comet assay to detect induced DNA breaks.

## Materials and Methods

### Reagents

Unless stated otherwise, all reagents were purchased from Sigma-Aldrich (St. Louis, MO, United States).

### Semen Samples

Sperm samples were obtained from sexually mature Piétrain boars (*n* = 11) hosted in a local farm operating under standard conditions for the production of commercial, artificial insemination doses (Servicios Genéticos Porcinos, S.L.; Roda de Ter, Barcelona, Spain). Since animals were not directly manipulated for the present study, no specific ethical approval was required.

Ejaculates were obtained using the gloved-hand technique, and samples were diluted 2:1 (v/v) in Vitasem long-term extender (Magapor, S.L.; Zaragoza, Spain). Upon arrival, an aliquot was used to evaluate sperm quality, and the remaining volume was stored at 17°C for 24 h and then cryopreserved (Yeste et al., 2014).

### Sperm Cryopreservation

Each semen sample was split into 50-mL aliquots and centrifuged at 2,400 × *g* and 17°C for 4 min. Pellets were suspended in β-lactose-egg yolk (LEY) media, containing 80% (v/v) lactose and 20% (v/v) egg yolk, reaching a concentration of 1.5 × 10^9^ sperm/mL, and cooled down to 5°C at a rate of −0.1°C/min. Afterward, the mixture was diluted in LEYGO medium (LEY medium supplemented with 6% v/v glycerol and 1.5% Orvus ES Paste; Equex STM, Nova Chemical Sales Inc., Scituate, MA, United States) to a final concentration of 1 × 10^9^ sperm/mL. At this point, samples were packaged in 0.5-mL straws (MiniTüb; Tiefenbach, Germany), and cryopreserved as follows: 6°C/min from 5 to −5°C (100 s); −39.82°C/min from −5 to −80°C (113 s); holding at −80°C for 30 s; and cooled at −60°C/min from −80 to −150°C (70 s). Finally, samples were stored in liquid nitrogen. Thawing was performed through immersion of cryopreserved straws in a water bath at 38°C for 15 s and subsequent dilution in three volumes of pre-warmed Beltsville Thawing Solution (BTS).

### Analysis of Sperm Motility Through Computer-Assisted Sperm Analysis (CASA)

Sperm motility was analyzed using a commercial computer-assisted system (Integrated Sperm Analysis System V1.0; Proiser SL, Valencia, Spain). First, samples were warmed at 38°C for 15 min and loaded onto a Makler chamber (Sefi-Medical Instruments, Haifa, Israel) previously warmed to 38°C. Different fields were captured at 100× magnification and at a rate of 25 frames/s, until 1,000 spermatozoa per assessment were counted. Three replicates were evaluated per sample. Parameters recorded were: percentages of progressively motile spermatozoa and total motile spermatozoa; curvilinear velocity (VCL; the instantaneously recorded sequential sperm progression along the whole trajectory; μm/s); straight-line velocity (VSL; the straight sperm trajectory per unit of time; μm/s); average path velocity (VAP; the mean sperm trajectory per unit of time; μm/s); linearity coefficient [LIN; (VSL/VCL) × 100; %]; straightness coefficient [STR; (VSL/VAP) × 100; %]; wobble coefficient [WOB; (VAP/VCL) × 100; %]; mean amplitude of lateral head displacement (ALH; the mean amplitude of the lateral oscillatory movement of the sperm head around the mean trajectory; μm) and frequency of head displacement (BCF; the number of sperm head lateral oscillatory movements around the mean trajectory per unit of time; Hz).

### Flow Cytometry

Sperm viability was evaluated through the assessment of membrane integrity using the LIVE/DEAD Sperm Viability Kit (Molecular Probes, Eugene, OR, United States), following the protocol of Garner and Johnson (1995). Briefly, sperm (1 × 10^6^ spermatozoa/mL) were incubated with SYBR-14 (final concentration: 100 nmol/L) for 10 min and then with Propidium Iodide (final concentration: 12 μmol/L) for 5 min at 38°C. Samples were evaluated using a Cell Laboratory QuantaSC^TM^ cytometer (Beckman Coulter; Fullerton, CA, United States) and were excited with an argon ion laser (488 nm) set at a power of 22 mW.

In flow cytometry dot-plots, three sperm populations were observed: (1) viable, green-stained sperm (SYBR-14^+^/PI^–^); (2) non-viable, red-stained sperm (SYBR-14^–^/PI^+^); (3) non-viable, both red- and green-stained sperm (SYBR-14^+^/PI^+^). The fourth dot population corresponded to unstained, non-sperm particles (SYBR-14^–^/PI^–^). Viable green-stained sperm were used to assess sperm viability, and SYBR-14 fluorescence spill over into FL3-channel was compensated (2.45%).

### Treatments to Induce Single and Double Strand DNA Breaks

For the generation of positive controls, five fresh and five cryopreserved samples were treated with H_2_O_2_ and DNAse I treatments prior to conducting alkaline or neutral Comet assays.

#### Generation of Single-Strand DNA Breaks Using Hydrogen Peroxide

For the generation of positive samples for alkaline Comet, sperm were first permeabilized with 0.25% Triton X-100 in PBS for 5 min and then washed twice in PBS. After washing, 100 × 10^6^ sperm were incubated with either 0.1% H_2_O_2_ (42.62 mM) or 0.01% H_2_O_2_ (4.26 mM) at room temperature for 30 min. After treatment, samples were washed three times in 5 mL PBS through centrifugation at 600 × *g* and room temperature for 5 min. A schematic diagram of the hydrogen peroxide treatments is depicted in Supplementary Figure 1.

#### Generation of Extensive DNA Damage, Including Both Single- and Double-Strand DNA Breaks

For the generation of positive samples for both types of DNA breaks, sperm samples were permeabilized using PBS supplemented with 0.25% Triton X-100 for 5 min. After two washings in PBS, 100 × 10^6^ sperm were incubated with 1 or 4 IU (enzymatic activity) of DNAse I (Thermo Fisher Scientific, Waltham, MA, United States) in a solution containing 10 mM MgCl_2_ and 10 mM CaCl_2_ at 37°C for 30 min. The reaction was stopped through adding 100 mM EDTA. A schematic overview of the DNAse I treatments is depicted in Supplementary Figure 1.

### Alkaline and Neutral Comet Assay

The Comet assay performed in neutral conditions was used for the analysis of double-strand DNA breaks, whereas the alkaline Comet was used for the assessment of extensive DNA damage consisting of single- and double-strand DNA breaks. The Comet assay was based on the protocol set by Casanovas et al. (2019) for humans, adapted to pig sperm. Supplementary Figure 2 schematizes the workflow for the performance of the Comet assay in the laboratory.

#### Sperm-Agarose Mixture and Lysis Steps

Sperm samples were diluted in PBS to a final concentration of 1 × 10^6^ spermatozoa/mL. In the meantime, an aliquot of 1% low melting point agarose was melted at 70°C for 10 min and subsequently incubated at 37°C for further 10 min. After warming sperm samples at 37°C, they were mixed with low melting point agarose at 1:2 ratio, leading to a final agarose concentration of 0.66%. Afterward, 6.5 μL of the mixture was dispensed onto two agarose pre-treated slides, one designated to the alkaline Comet and the other designated to the neutral Comet; slides were then covered with 8-mm diameter round coverslips. Then, agarose was jellified on a cold plate at 4°C for 5 min and coverslips were gently removed. Slides were incubated in a first lysis solution containing 0.8 M Tris–HCl, 0.8 M DTT and 1% SDS (pH 7.5) for 30 min, and then in a second lysis solution containing 0.4 M Tris–HCl, 0.4 M DTT, 50 mM EDTA, 2 M NaCl and 1% Tween20 (pH 7.5) for 30 min.

#### Prolonged Additional Lysis Step

After the aforementioned lysis steps, and according to the previous results obtained in pig sperm (Ribas-Maynou et al., 2021a), slides were incubated in a third lysis solution that contained proteinase K (0.4 M Tris–HCl, 0.4 M DTT, 50 mM EDTA, 2 M NaCl, 1% Tween20 and 100 μg/mL Proteinase K; pH 7.5). This third lysis treatment was performed for different incubation times: 0, 30, and 180 min. After this treatment, slides were washed in a neutral pH solution (0.4 M Tris–HCl; pH 7.5) for 10 min.

#### Electrophoresis

Electrophoresis steps were differentially conducted depending on the Comet test. Slides intended to the alkaline Comet were first denatured in a cold alkaline solution (0.03 M NaOH, 1 M NaCl) for 5 min, and then electrophoresed at 1 V/cm for 4 min in an alkaline buffer (0.03 M NaOH; pH 13).

For slides designated to the neutral Comet, electrophoresis was performed in TBE buffer (0.445 M Tris–HCl, 0.445 M Boric acid and 0.01M EDTA; pH 8) at 1 V/cm for 4 min; slides were subsequently washed in 0.9% NaCl solution for 2 min.

#### Neutralization, Dehydration, and Staining

After electrophoresis, all slides were incubated in neutralization solution (0.4 M Tris–HCl; pH 7.5) at room temperature, and dehydrated in an ethanol series of 70, 90, and 100%. Finally, slides were dried horizontally, stained with 5 μL of 1 × Safeview DNA stain (NBS Biologicals, Huntingdon, United Kingdom) and covered with 20 × 20 coverslips.

#### Imaging

Samples were visualized under a Zeiss Imager Z1 epifluorescence microscope (Carl Zeiss AG, Oberkochen, Germany). Captures of at least 100 sperm per sample were performed at 100 × magnification using Axiovision 4.6 software (Carl Zeiss AG, Oberkochen, Germany), at a resolution of 1,388 × 1,040 pixels. Exposure time was adapted to avoid overexposure of Comet heads compared to Comet tails; Comet heads were oriented toward the left side and Comet tails toward the right side of the image.

#### Analysis of Comet Images

Comet analysis was performed using the open-access CometScore v2.0 software (RexHoover^1^), which captures the fluorescence intensity of Comet heads and Comet tails. All images were evaluated automatically, adjusting the background intensity to correctly visualize Comet heads and tails. After automatic analysis, a manual review of the analyzed Comets was performed with the aim of: (a) eliminating captures not corresponding to Comets (i.e., debris); (b) removing overlapping Comets; or (c) correcting the head and tail detection for those Comets that were misanalysed. When the final number of correctly analyzed Comets was less than 50, more images from slides were captured and the process was repeated until obtaining at least 50 correctly analyzed images.

Among the high variety of parameters that the analysis offers, tail DNA (TD) and olive tail moment (OTM) were chosen as they have been shown to be the most informative in previous studies (Langie et al., 2015; Simon et al., 2017a). TD is a relative measurement of the amount of DNA in the Comet tail in relation to the total DNA amount, expressed in percentage and calculated as: (tail intensity)/(comet intensity).

Olive tail moment is a more complex measurement that takes into account the amount of TD, but also the differences of optical intensities between the Comet head and the Comet tail. This parameter was calculated as: (tail mean intensity – head mean intensity) × TD/100.

### Analysis of Pig Sperm Samples Using the Comet Assay

In order to describe the incidence of single- and double-strand DNA breaks in pig sperm, three incubation times with lysis buffer (0, 30, and 180 min) were tested together with alkaline and neutral Comet assays. Semen samples from 11 different boars were used.

### Analysis of the Effects of the Third Lysis Solution on DNA Breaks

In order to determine if the incubation with the third lysis solution caused DNA damage in sperm samples, we performed an additional experiment. Five sperm samples were pre-incubated in the third lysis solution (L3), and DNA fragmentation was subsequently analyzed through alkaline Comet, neutral Comet and SCD tests.

### Sperm Chromatin Dispersion Test

Sperm chromatin dispersion test was conducted through the following procedure. First, samples were washed twice in PBS and diluted to 2 × 10^6^ sperm/mL in PBS. Thereafter, sperm samples were mixed (1:2) with low melting point agarose at 37°C, and 6.5 μL of the mixture were allowed to jellify onto an agarose-pretreated slide, at 4°C for 5 min. Then, the slide was incubated in the three lysis solutions (30 min for Lysis 1, 30 min for Lysis 2, and 180 min for lysis 3) that allow complete chromatin decondensation (Ribas-Maynou et al., 2021a). After the lysis, the slide was washed in distilled water, neutralized in neutralization solution (0.4 M Tris–HCl; pH 7.5), dehydrated in ethanol series (70, 90, and 100%) and dried in horizontal position. The analysis of the halo area, analyzed using ImageJ was performed as a quantitative parameter for DNA breaks.

### Statistical Analyses

Data were analyzed using the Statistics Package for Social Sciences (SPSS, Ver. 25.0; IBM Corp.; Armonk, NY, United States). Graphs were compiled with GraphPad Prism 8.0 software (GraphPad, San Diego, CA, United States).

#### Determination of Percentages of Sperm With Fragmented DNA

Percentages of sperm with fragmented DNA were determined with receiver operating characteristic (ROC) curves. This procedure, which allowed setting the threshold for considering a spermatozoon as bearing fragmented DNA, was performed after incubating sperm with proteinase K for 0, 30, and 180 min, and in both fresh and cryopreserved samples. For alkaline Comet, sperm treated with 0.01% H_2_O_2_ and with 0.1% H_2_O_2_ were used as positive controls, whereas non-treated sperm were used as the negative ones. For neutral Comet, sperm treated with DNAse I (1 IU and 4 IU) were used as positive controls and non-treated sperm were used as negative controls. After comparing the area under the curve (AUC) obtained for TD and OTM, the percentage of sperm with fragmented DNA obtained with the parameter showing the highest AUC was calculated for each sample. The confidence level was set at 0.95.

#### Determination of Differences Among Groups and Correlations

In order to assess the effect of different treatments, the mean of sperm TD and the mean of sperm OTM for each sample and treatment were recorded. Fresh and cryopreserved samples were analyzed separately. Normal distribution and homogeneity of variances were checked through Shapiro–Wilk and Levene tests. Since, even after linear transformation [log(*x*), arcsine √x, √x], data did not fit with parametric assumptions, Scheirer–Ray–Hare and Mann–Whitney *U* tests were run to determine differences between groups.

Correlations were calculated using the Spearman test. In all cases, the level of significance was set at *P* ≤ 0.05.

## Results

### Olive Tail Moment and TD Represent a Quantitative Measurement of DNA Breaks

Among the wide variety of parameters that Comet analysis offers, we tested whether OTM and TD could quantitatively measure the incidence of DNA breaks in positive controls prepared with H_2_O_2_ and DNAse. Table 1 shows mean and standard deviation (SD) of OTM and TD for the different treatments, following alkaline and neutral Comet assays, in both fresh and cryopreserved samples.

**TABLE 1:** Olive tail moment and tail DNA parameters showing the amount of sperm DNA breaks detected in sperm cells after different H_2_O_2_ and DNAse I treatments in different proteinase K conditions.

	Comet Olive tail			Comet tail DNA		
	Proteinase K 0 min	Proteinase K 30 min	Proteinase K 180 min	Proteinase K 0 min	Proteinase K 30 min	Proteinase K 180 min
**(A) Fresh – alkaline comet**						
Control	2.95 ± 2.33	10.44 ± 6.00*	28.55 ± 3.59*^,¶^	14.23 ± 5.19	33.70 ± 15.47*	69.40 ± 14.62*^,¶^
H_2_O_2_ 0.01%	11.89 ± 9.84	24.83 ± 12.31	38.10 ± 7.32*	31.13 ± 15.23	54.89 ± 19.79	91.65 ± 7.38*^,¶,a^
H_2_O_2_ 0.1%	14.22 ± 6.11^a^	23.64 ± 17.99	46.38 ± 3.45*^,¶,a^	38.47 ± 10.96^a^	55.21 ± 26.08	95.63 ± 0.94*^,¶,a^
DNAse I – 1U	8.77 ± 3.28^a^	15.66 ± 9.85	34.90 ± 7.04*^,¶^	29.03 ± 5.64^a^	44.53 ± 17.03	86.16 ± 8.54*^,¶,a^
DNAse I – 4U	10.04 ± 7.91^a^	18.06 ± 8.93	38.28 ± 7.59*^,¶,a^	31.47 ± 10.59^a^	47.36 ± 19.72	85.58 ± 5.73*^,¶,a^
**(B) Fresh – neutral comet**						
Control	1.06 ± 0.57	1.69 ± 1.24	1.50 ± 0.72*	16.37 ± 2.96	15.18 ± 2.70	12.99 ± 1.35*
H_2_O_2_ 0.01%	0.80 ± 0.12	1.74 ± 1.58*	2.23 ± 0.95*	14.67 ± 2.37	14.13 ± 2.27	15.24 ± 2.27
H_2_O_2_ 0.1%	1.00 ± 0.27	1.49 ± 0.98	1.53 ± 0.52	16.06 ± 2.08	13.45 ± 2.51	11.97 ± 1.91*
DNAse I – 1U	14.12 ± 11.15^a^	10.47 ± 6.17^a^	25.69 ± 14.97^a^	32.24 ± 11.24^a^	25.12 ± 6.23^a^	39.29 ± 17.06^a^
DNAse I – 4U	18.05 ± 6.27^a^	17.06 ± 8.73^a^	29.55 ± 10.94^a^	29.24 ± 7.87^a^	32.46 ± 11.51^a^	46.41 ± 12.71^¶,*a*^
**(C) Cryopreserved – alkaline comet**						
Control	13.18 ± 7.86	22.80 ± 12.87	27.14 ± 9.01*	36.72 ± 9.62	55.65 ± 23.43	73.59 ± 17.59*
H_2_O_2_ 0.01%	28.19 ± 14.88	32.86 ± 13.81	32.81 ± 20.61	58.91 ± 20.82	73.65 ± 17.97	70.26 ± 33.73*
H_2_O_2_ 0.1%	26.78 ± 15.02	32.05 ± 14.69	38.40 ± 10.29	57.11 ± 17.62^a^	73.60 ± 16.20	91.62 ± 10.47^¶,*a*^
DNAse I – 1U	6.58 ± 6.47	13.29 ± 12.22	27.98 ± 13.16*^,¶^	26.58 ± 11.02	36.77 ± 9.94	68.47 ± 18.55*^,¶^
DNAse I – 4U	12.91 ± 12.07	15.10 ± 14.23	30.93 ± 16.57*^,¶^	37.92 ± 21.65	43.09 ± 6.51	78.88 ± 11.34*^,¶^
**(D) Cryopreserved – neutral comet**						
Control	0.79 ± 0.18	0.98 ± 0.12	1.24 ± 0.23*	13.47 ± 2.04	13.27 ± 2.55	14.74 ± 1.35
H_2_O_2_ 0.01%	0.90 ± 0.04	1.17 ± 0.52	1.48 ± 0.58	16.02 ± 2.21	13.13 ± 2.44	13.81 ± 3.26
H_2_O_2_ 0.1%	0.88 ± 0.09	0.96 ± 0.07	5.13 ± 9.09*^,¶^	16.95 ± 3.40	13.38 ± 2.21	28.42 ± 33.42
DNAse I – 1U	10.94 ± 20.89^a^	19.85 ± 16.45*^,a^	28.30 ± 10.65*	21.11 ± 7.74	31.17 ± 11.67	44.99 ± 11.62*
DNAse I – 4U	20.24 ± 22.96	24.57 ± 17.26^a^	45.87 ± 12.46*^,¶,a^	28.43 ± 14.43^a^	42.49 ± 12.37^a^	65.81 ± 15.74*^,¶,a^

*Differences compared to 0 min of proteinase K in similar treatment (*p* < 0.05). ^¶^Differences compared to 30 min of proteinase K in similar treatment (*p* < 0.05). ^*a*^Differences compared to control in similar proteinase K treatment (*p* < 0.05).

Incubation with the two concentrations of H_2_O_2_ increased alkaline Comet parameters, in both fresh and cryopreserved sperm (Table 1A,C). Following the additional lysis step of fresh sperm with proteinase K, these increases were statistically significant for the highest H_2_O_2_ concentration in OTM and for the two H_2_O_2_ concentrations in TD (*P* < 0.05). When cryopreserved samples were used, statistical increments of the alkaline Comet were observed at the highest H_2_O_2_ concentration (0.1%) for TD (*P* < 0.05).

Treatments with DNAse I increased neutral Comet parameters in both fresh and cryopreserved sperm, and those of the alkaline Comet in fresh samples (Table 1).

### The Additional Lysis Step Leads to Higher Chromatin Decondensation

**FIGURE 1:**
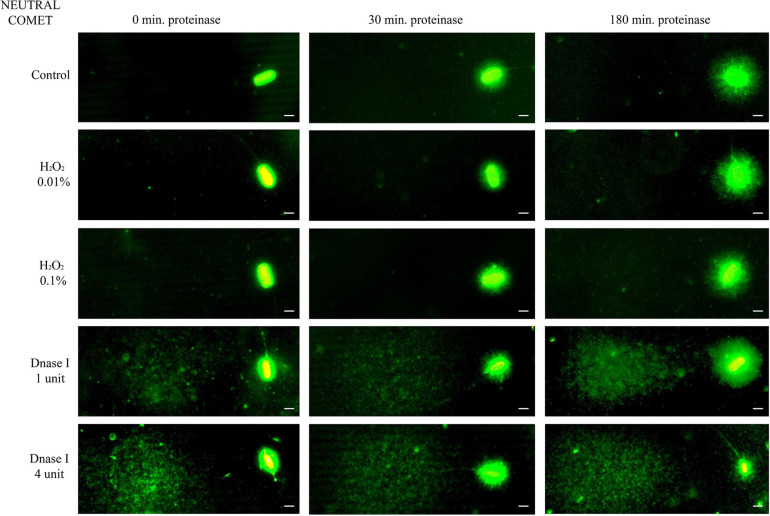
Representative images of the neutral Comet after incubation with the additional lysis solution containing proteinase K (columns) for different times and after treating samples with H_2_O_2_ and DNAse I (rows). Scale bar: 5 μm.

Further incubation with proteinase K for 30 min and 180 min led to more decondensed chromatin; this was clearly observed in sperm with non-fragmented DNA following the neutral Comet assay (Figure 1, control sperm). Moreover, fragmented comets depicted a higher percentage of DNA released from the sperm nucleus. The higher amount of DNA resulting from this complete decondensation was visualized in the Comet tail after electrophoresis (Figure 1, DNAse I treatments).

**FIGURE 2:**
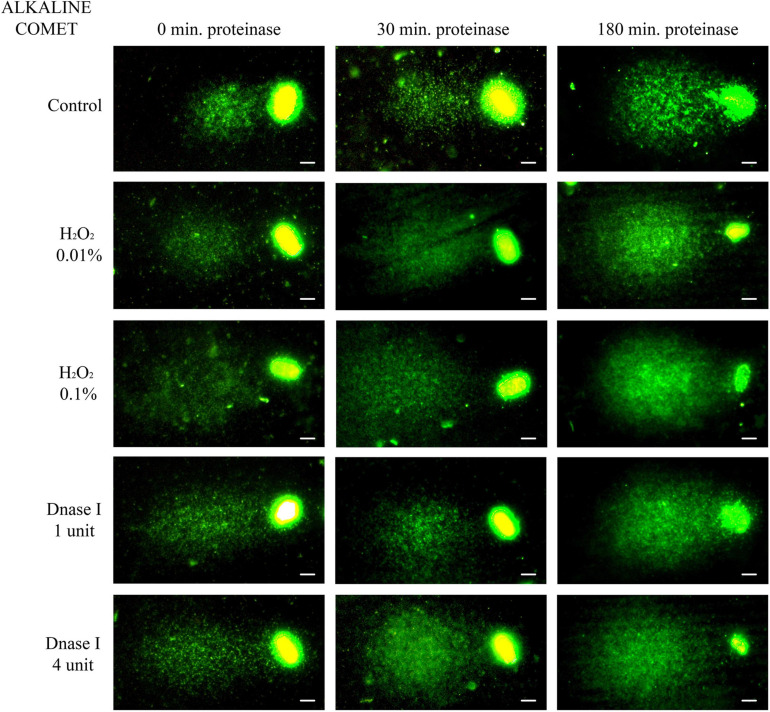
Representative images of the alkaline Comet after incubation with the additional lysis solution containing proteinase K (columns) for different times and after treating samples with H_2_O_2_ and DNAse I (rows). Scale bar: 5 μm.

Non-treated alkaline comets always depicted a Comet tail, since alkaline-labile regions are known to break up when alkaline DNA unwinding is performed (Ribas-Maynou et al., 2012a; Cortés-Gutiérrez et al., 2014). Comparison of different incubation times in the lysis solution containing proteinase K indicated that longer treatment led to a significant increase in both OTM and TD (*P* < 0.05, Figure 2 and Table 1).

### Higher Chromatin Decondensation Leads to Higher Sensitivity in the Detection of DNA Breaks

**FIGURE 3:**
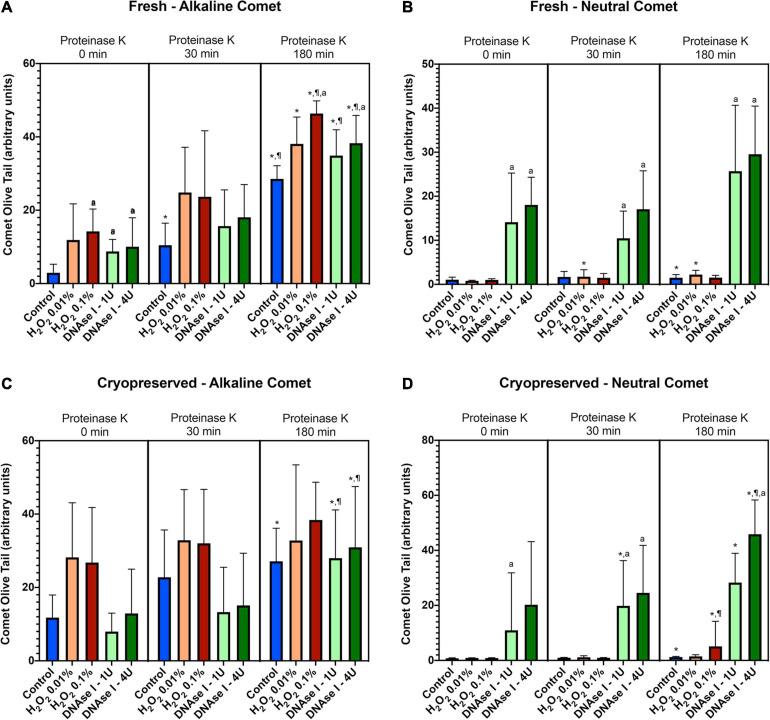
Comet olive tail moment (OTM) showing the amount of DNA breaks detected in sperm after different proteinase K treatments. Panel **(A)** shows OTM for alkaline Comet in fresh samples. Panel **(B)** depicts OTM for neutral Comet in fresh samples. Panel **(C)** shows OTM for alkaline Comet in cryopreserved samples. Panel **(D)** depicts OTM for neutral Comet in cryopreserved samples. (*) Statistical differences compared to the similar treatment in 0 min of proteinase K (*P* < 0.05). (¶) Statistical differences compared to the similar treatment in 30 min of proteinase K (*P* < 0.05). (a) Statistical differences compared to the control of the same proteinase K treatment (*P* < 0.05).

To test whether the additional lysis step containing proteinase K resulted in higher sensitivity for the detection of DNA breaks following neutral and alkaline Comet assays, we compared OTM and TD after different incubation times (Figure 3 and Table 1). The additional lysis step including proteinase K (180 min) led to an increase of both OTM and TD in control samples analyzed with the alkaline Comet (*P* < 0.05), but not in samples analyzed with the neutral variant (*P* > 0.05).

Regarding the alkaline Comet, incubation with the additional lysis step led to an increase in the amount of DNA breaks, depicted as higher OTM and TD, when treatments with H_2_O_2_ or DNAse I were performed. For fresh samples, these differences were statistically significant in OTM and TD for all H_2_O_2_ and DNAse I incubations, when 0 min and 180 min of incubation were compared (*P* < 0.05, Figure 3A and Table 1). As far as cryopreserved samples are concerned, statistical differences in OTM were found in incubations with the two concentrations of DNAse I comparing 180 min of lysis with 0 or 30 min (*P* < 0.05, Figure 3C and Table 1). TD showed differences between different proteinase K treatments for both H_2_O_2_ (0.1%) and DNAse I (1 IU and 4 IU) treatments (*P* < 0.05, Table 1).

The neutral Comet showed an increase of OTM in DNAse I–treated samples, this variable being significantly different when incubation for 180 min was compared with 0 min and 30 min, in cryopreserved sperm treated with either 1 IU or 4 IU DNAse (*P* < 0.05; Figures 1, 3D). This increase was also observed in TD after treating samples with 4 IU DNAse I (Table 1; *P* < 0.05).

### Olive Tail Moment Is More Suitable Than TD to Identify Sperm With Fragmented and Non-fragmented DNA

In order to determine the percentage of sperm with fragmented DNA for each sample, ROC curves were calculated, comparing each positive control with its corresponding negative counterpart in every lysis condition. For alkaline Comet, we used the two H_2_O_2_ treatments as positive controls; the AUC including the 95% of confidence interval is depicted in Table 2A, for both OTM and TD. For neutral Comet, we used the two DNAse treatments as positive controls; the AUC including the 95% of confidence interval is depicted in Table 2B, for both OTM and TD.

**TABLE 2:** Area under the curve for the determination of sperm cells with fragmented DNA in each proteinase K condition.

Lysis containing proteinase K		0 min		30 min		180 min	
		AUC	95% CI	AUC	95% CI	AUC	95% CI
**(A) Olive tail moment**							
Fresh	Alkaline Comet	0.708	(0.668–0.749)	0.758	(0.705–0.811)	0.890	(0.859–0.920)
Fresh	Neutral Comet	0.884	(0.851–0.918)	0.682	(0.649–0.716)	0.807	(0.776–0.839)
**(B) Tail DNA**							
Fresh	Alkaline Comet	0.732	(0.693–0.771)	0.746	(0.693–0.800)	0.985	(0.971–0.999)
Fresh	Neutral Comet	0.804	(0.762–0.845)	0.611	(0.575–0.647)	0.788	(0.755–0.822)

Since for most parameters, especially after 30 and 180 min of incubation with proteinase K, the AUC was higher for OTM than for TD, cut-off OTM values were calculated to set a threshold of sperm DNA fragmentation. Cut-off values for alkaline Comet in fresh samples were 2.17, 11.06, and 36.29 for 0, 30, and 180 min of additional lysis, respectively. Cut-off values neutral Comet in fresh samples were 3.62, 1.26, and 2.80 for 0, 30, and 180 min of additional lysis, respectively.

### Determination of the Percentage of Fragmented Comets Requires a Precise Determination of Positive Controls in Each Condition

**FIGURE 4:**
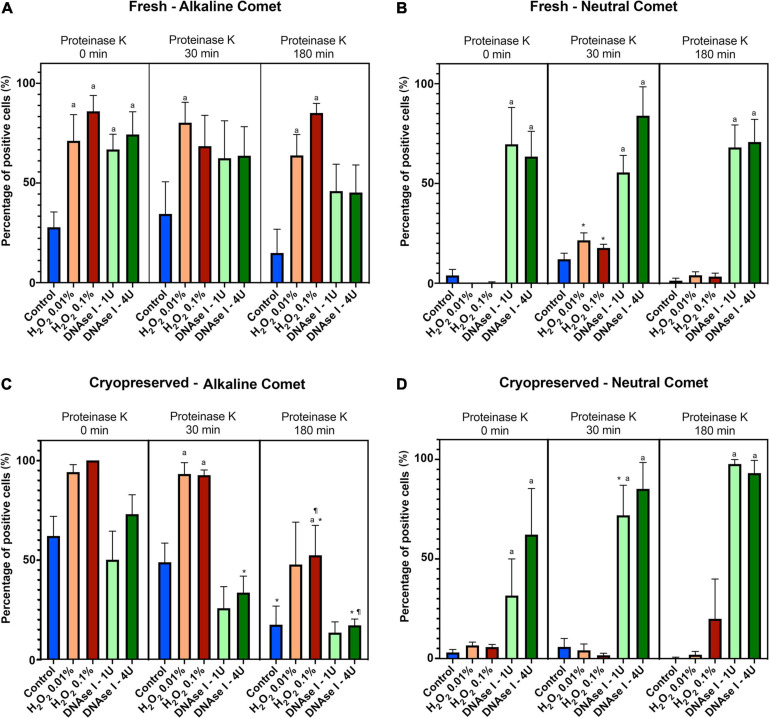
Percentage of sperm DNA fragmentation (% SDF) after different Proteinase K treatments. Panel **(A)** shows % SDF for alkaline Comet in fresh samples. Panel **(B)** depicts % SDF for neutral Comet in fresh samples. Panel **(C)** shows % SDF for alkaline Comet in cryopreserved samples. Panel **(D)** depicts % SDF for neutral Comet in cryopreserved samples. (*) Statistical differences compared to the similar treatment in 0 min of proteinase K (*P* < 0.05). (¶) Statistical differences compared to the similar treatment in 30 min of proteinase K (*P* < 0.05). (a) Statistical differences compared to the control of the same proteinase K treatment (*P* < 0.05).

Using the cut-off values defined for OTM following alkaline and neutral Comet assays for each time of incubation in the additional lysis solution, we could calculate the percentages of sperm with fragmented DNA following each treatment (Table 3 and Figure 4). DNAse I treatments showed a significant (*P* < 0.05) increase in the percentage of sperm with fragmented DNA following the neutral Comet in both fresh and cryopreserved samples, except for 0 min of incubation with proteinase K (Figures 4B,D). For alkaline Comet, treatments with H_2_O_2_ led to an increase in the percentage of sperm with fragmented DNA in both fresh and cryopreserved samples (*P* < 0.05, Figure 4), except for 0 min of incubation with proteinase K treatment, where only a tendency was observed (*P* = 0.068). However, these latter increases did not show statistical differences in cryopreserved samples (*P* > 0.05, Figure 4C).

**TABLE 3:** Percentage of sperm cells with DNA damage after hydrogen peroxide and DNAse treatments in different proteinase K conditions.

	Percentage of positive cells		
	Proteinase K 0 min	Proteinase K 30 min	Proteinase K 180 min
**Fresh – alkaline comet**			
Control	30 ± 22%	35 ± 36%	15 ± 24%
H_2_O_2_ 0.01%	71 ± 29%^a^	80 ± 23%^a^	64 ± 23%^a^
H_2_O_2_ 0.1%	86 ± 18%^a^	69 ± 35%	85 ± 11%^a^
DNAse I – 1U	67 ± 17%^a^	62 ± 42%	46 ± 30%
DNAse I – 4U	74 ± 26%^a^	64 ± 33%	45 ± 31%
**Fresh – neutral comet**			
Control	6 ± 12%	14 ± 13%	1 ± 3%
H_2_O_2_ 0.01%	0 ± 0%	23 ± 16%*	4 ± 4% ^¶^
H_2_O_2_ 0.1%	0 ± 1%	18 ± 4%*	3 ± 4% ^¶^
DNAse I – 1U	70 ± 41%^a^	56 ± 19%^a^	68 ± 25%^a^
DNAse I – 4U	63 ± 25%^a^	84 ± 32%^a^	71 ± 25%^a^
**Cryopreserved – alkaline comet**			
Control	62 ± 22%	48 ± 21%	17 ± 20%*
H_2_O_2_ 0.01%	94 ± 8%	93 ± 13%^a^	48 ± 47%
H_2_O_2_ 0.1%	100 ± 0%	93 ± 6% ^a^	52 ± 13%^*a,¶*,^ *
DNAse I – 1U	50 ± 32%	26 ± 13%	14 ± 12%
DNAse I – 4U	73 ± 22%	34 ± 19%*	17 ± 7%*^¶^
**Cryopreserved – neutral comet**			
Control	0 ± 0%	19 ± 9%*	0 ± 1%^¶^
H_2_O_2_ 0.01%	0 ± 0%	19 ± 7%*	3 ± 3% ^¶^
H_2_O_2_ 0.1%	0 ± 0%	15 ± 1%*	20 ± 20%
DNAse I – 1U	9 ± 15%	70 ± 27%^*a*,^*	97 ± 5%^*a*,^*
DNAse I – 4U	50 ± 50%	83 ± 26%^a^	99 ± 1% ^a^

*Differences compared to 0 min of proteinase K in similar treatment (*p* < 0.05). ^¶^Differences compared to 30 min of proteinase K in similar treatment (*p* < 0.05). ^*a*^Differences compared to control in similar proteinase K treatment (*p* < 0.05).

Differences between 0 and 180 min of incubation with proteinase K were observed in controls and samples treated with 0.1% H_2_O_2_ and 4 IU DNAse (*P* < 0.05) in the case of the alkaline Comet, and in samples treated with 1 IU DNAse in the case of the neutral variant (*P* < 0.05).

### Incidence of DNA Breaks in the Alkaline Comet Is Related to Sperm Motility Parameters

**FIGURE 5:**
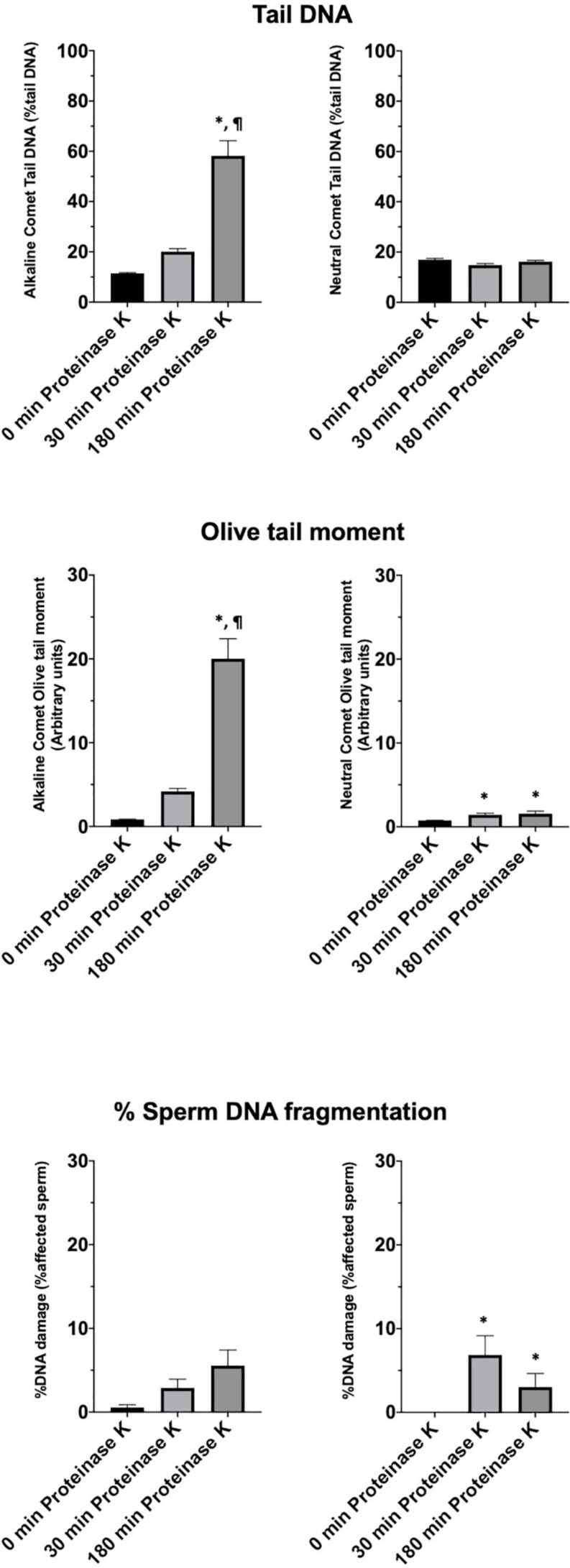
Tail DNA, olive tail moment and percentage of affected sperm in 11 boars using alkaline and neutral Comet assays after different proteinase K treatments. (^∗^) Statistical differences compared to the similar treatment in 0 min of proteinase K (*P* < 0.05). (¶) Statistical differences compared to the similar treatment in 30 min of proteinase K (*P* < 0.05).

Motility and viability data for the 11 analyzed boars are displayed in Table 4. Using the settings established for both Comet assays, we analyzed a subset of cryopreserved pig sperm samples to characterize: (a) the incidence of single- and double-strand DNA breaks, measured through both TD and OTM; and (b) the incidence of sperm with fragmented DNA analyzed using the ROC curves defined before. Results for 11 boars are shown in Figure 5. Following the alkaline Comet, differences in the incidence of DNA damage were observed for TD and OTM (*P* < 0.05), but not for the percentage of sperm with fragmented DNA, suggesting that this parameter could be independent from chromatin decondensation. Regarding the neutral Comet, both TD and OTM showed differences after chromatin decondensation with proteinase K (*P* < 0.05).

**TABLE 4:** Sperm viability and motility parameters from samples used to analyze sperm DNA fragmentation using alkaline and neutral Comet assay.

	Viability	Motility		Velocity				Velocity							
	% Viable	Total motility (%)	Progressive motility (%)	Fast (%)	Medium (%)	Slow (%)	Static (%)	VCL	VSL	VAP	LIN	STR	WOB	ALH	BCF
1	89.17	60.74	43.72	34.29	17.21	9.24	39.26	55.17	34.19	46.13	61.97	73.89	83.65	1.95	7.75
2	79.49	59.92	48.55	32.32	19.62	7.98	40.08	58.74	43.55	51.36	73.87	84.66	87.21	1.87	7.83
3	84.02	57.65	49.05	34.43	17.40	5.82	42.35	54.54	40.30	48.49	73.91	83.13	88.91	1.77	7.66
4	78.69	92.36	49.84	89.49	2.37	0.50	7.64	96.94	33.67	69.20	34.76	48.70	71.45	3.46	7.53
5	86.55	87.85	41.70	57.61	20.05	10.18	12.15	69.93	25.14	54.87	35.99	45.84	78.49	2.51	6.95
6	75.79	66.05	42.87	37.33	17.12	11.59	33.95	54.80	35.29	46.15	64.50	76.54	84.25	1.94	7.62
7	64.69	37.49	15.74	3.52	9.15	18.85	68.48	26.39	15.12	19.88	57.54	75.87	75.68	1.13	6.11
8	84.74	82.41	65.68	64.74	14.53	3.14	17.59	63.51	33.83	50.27	53.41	67.71	79.01	2.34	7.69
9	73.11	64.16	38.80	17.04	27.62	19.50	35.84	36.22	22.01	29.15	61.25	75.75	80.69	1.53	8.30
10	80.97	68.31	49.92	45.80	14.33	8.18	31.69	60.34	35.03	50.75	58.13	69.27	83.95	2.07	7.89
11	73.62	49.47	29.15	35.20	8.79	5.49	50.53	68.67	23.07	42.32	33.64	54.56	61.75	2.76	7.82

On the other hand, samples incubated with proteinase K for 180 min showed, following the alkaline Comet, negative correlations between TD and progressive motility (Rs = −0.673; *P* = 0.023), and between TD and VSL (Rs = −0.664; *P* = 0.026). No correlation between the other motility parameters and DNA fragmentation was observed.

### The Third Lysis Solution Does Not Produce Additional DNA Breaks

The additional experiment performed to elucidate if the third lysis solution caused DNA breaks revealed that neither the OTM value of alkaline (26.01 ± 9.98 and 26.47 ± 10.22 for untreated and L3-treated samples, respectively; *P* = 0.586) or neutral Comet (3.32 ± 12.97 and 3.50 ± 13.14, for untreated and L3-treated samples, respectively; *P* = 0.933), nor the halo area of SCD test (1586.52 ± 367.73 and 1590.99 ± 337.71, for untreated and L3-treated samples, respectively; *P* = 0.596) differed between treated and untreated samples.

## Discussion

The Comet assay is a very used technique in multiple research fields (Faust et al., 2004; Santos et al., 2015; Gajski et al., 2019). Despite the fact that no standardization has been achieved across laboratories, the Comet assay is recognized as a reliable method for the study of DNA integrity, and has been properly applied to sperm cells (Singh et al., 1988; Simon et al., 2010; Albert et al., 2016). Recent meta-analyses conducted in human sperm indicated that direct methods for the evaluation of sperm DNA fragmentation are better correlated with IVF and ICSI outcomes than the indirect ones (Simon et al., 2017b; Deng et al., 2019). Remarkably, not only did previously research show that the number of DNA breaks and the percentages of sperm with fragmented DNA determined by the Comet assay are linked to male infertility (Lewis and Simon, 2010; Simon et al., 2011; Ribas-Maynou et al., 2012a), but they also demonstrated that specific types of DNA breaks underlie miscarriage during the first trimester (Ribas-Maynou and Benet, 2019; Agarwal et al., 2020; Coban et al., 2020). In farm animals, such as pigs, while sperm fertilizing ability is mainly estimated through conventional semen analysis, only few studies used sensitive DNA damage methods to analyze single- and double-strand DNA breaks (Hu et al., 2008; Fraser et al., 2010, 2014; Pérez-Llano et al., 2010; Enciso et al., 2011). In the present study, we found that complete chromatin decondensation of pig sperm is required for a more sensitive detection of single- and double-strand sperm DNA breaks through Comet assays. Moreover, we established OTM as a reliable predictor of the amount of DNA breaks, and we defined thresholds for sperm with fragmented and non-fragmented DNA. Following this, we could evaluate the inherent variability of semen samples from 11 boars comparing the different lysis solutions tested, and investigate whether differences in genetic integrity were related to sperm quality.

The demonstration, in this study, that complete chromatin decondensation is needed to determine the extent of DNA damage properly, is important for further studies using the Comet assay in porcine sperm. Complete chromatin decondensation through a specific lysis solution has previously been demonstrated in a recent work from our research group (Ribas-Maynou et al., 2021a) and is here evidenced in sperm with non-fragmented DNA (Figure 1). In effect, our findings indicate that incubation of pig sperm with a third lysis buffer containing proteinase K leads to higher chromatin decondensation, since larger haloes observed following the neutral Comet were more similar to those found in human sperm after lysis treatment (Ribas-Maynou et al., 2012a, 2021a; Casanovas et al., 2019). The alkaline Comet, in contrast, did not show such clear outcomes, as alkaline labile regions break up during DNA unwinding and, therefore, all sperm with non-fragmented DNA (control) presented DNA damage (Figure 2). Interestingly, this basal alkaline-induced DNA damage showed a ninefold increase (in OTM) when the additional third lysis treatment was applied for 180 min. Two hypotheses could explain this result. The first one would be that this third lysis solution causes new DNA breaks; the second would be that the higher chromatin decondensation induced by this third lysis treatment uncovers DNA breaks that are not apparent when chromatin is not completely decondensed.

Recently, we demonstrated that additional lysis containing proteinase K is required to completely decondense sperm chromatin in species that only contain protamine 1, such as pigs and cattle (Ribas-Maynou et al., 2021a). However, this additional lysis did not cause any change in species that contain both protamine 1 and protamine 2, such as humans, horses and donkeys (Ribas-Maynou et al., 2021a). As far as the first hypothesis is concerned, the use of other methods to determine DNA damage, such as TUNEL or SCD in sperm from species that only contain P1 could also be limited by chromatin condensation. Therefore, addressing whether or not does this third lysis solution cause DNA damage needs to envisage what happens in species that, like humans, horses and donkeys, contain both P1 and P2. In that case, the use of the SCD test in these three species revealed no changes in the halo/core ratio following incubation with the third lysis solution for 0, 30, or 180 min (Ribas-Maynou et al., 2021a). Additional experiments conducted herein showed that pre-incubation with the third lysis solution did not lead to further DNA damage measured through Comet and SCD tests. These results would thus rebut the first hypothesis. Furthermore, another work advised that enhanced protein depletion is necessary to evidence hybridization signal of alkaline labile sites in sperm from pig and sheep (Cortés-Gutiérrez et al., 2008), which is aligned with our findings. This suggests that alkaline labile regions may have higher incidence in the pig genome and they can only be completely detected when chromatin is fully decondensed. This specific requirement of complete chromatin decondensation could contribute to explain the discrepancies found between species by Cortés-Gutiérrez et al. (2008), who reported that whereas pig sperm show lower abundance of alkaline-labile sites than pig leukocytes, sheep sperm have higher alkaline-labile regions than their leukocyte counterparts. In humans, previous studies suggested that alkaline-labile regions present higher incidence in sperm than in somatic cells (Fernández et al., 2000; Muriel et al., 2004). Since the higher chromatin decondensation achieved by our prolonged lysis protocol with proteinase K showed five times more damaged DNA in the control alkaline Comet, we advise that re-analysis of alkaline-labile sites should be performed with more enhanced lysis solutions to better understand these previous results.

In the present study, sperm were incubated with H_2_O_2_ as a method to induce single-strand DNA breaks, which are produced through reaction of oxygen free radicals with both nitrogenized DNA bases and sugar backbones (Rueff et al., 1993; Enciso et al., 2009; Villani et al., 2010). Samples were also incubated with DNAse I supplemented with Mg^2+^ as a method to induce single- and double-strand DNA breaks. Due to the fact that ROS, which include H_2_O_2_, are small molecules with a free oxygen, they can diffuse into sperm nuclear DNA and exert a genotoxic effect (Aitken and De Iuliis, 2010; Villani et al., 2010). On the contrary, DNAses are proteins that cannot penetrate highly condensed protamine-regions and, therefore, they are sterically deprived from causing breaks to DNA condensed in toroids; thus, the sperm chromatin structure limits the activity of these enzymes (Ward, 2010). It is worth noting that the amount of DNA damage induced by H_2_O_2_ was higher than that induced by DNAse I (Table 1 and Figure 3), a fact that could be attributed to this sterical limitation. In addition, DNAse I treatments caused an increase of double-strand DNA breaks, detected through the neutral Comet, supporting that TD and OTM are a direct measure of sperm DNA breaks. While these Comet assay parameters are related to DNA damage in somatic cells and to male infertility in human sperm (Simon et al., 2013, 2017a), most clinical studies use the percentage of sperm with fragmented DNA as a Comet outcome (Bungum et al., 2004; Fernández et al., 2005; Thomson et al., 2011; Ribas-Maynou et al., 2012a; Sivanarayana et al., 2014).

On the other hand, we observed that alkaline and neutral Comet assays were able to detect a higher amount of DNA breaks when complete chromatin decondensation was achieved following additional incubation with proteinase K. Whilst these effects were found in all samples, the clearest results were observed in fresh sperm, as cryopreservation is known to induce DNA breaks and fresh samples depart from lower basal DNA fragmentation (Hu et al., 2008; Fraser et al., 2014; Ribas-Maynou et al., 2014). To the best of our knowledge, no previous study has investigated how DNA breaks induced by H_2_O_2_ and DNAse in pig sperm are detected following alkaline and neutral Comet assays. A study conducted in 2011 performed similar inductions, but the use of the two-tailed Comet assay did not allow the authors to obtain the amount of fragmented DNA; rather they could only report the percentage of sperm with increased damage (Enciso et al., 2011). Moreover, other studies using the Comet assay in pig sperm only performed the neutral variant, which solely detects double-strand breaks (Fraser et al., 2010, 2014). Thus, the current study is the first one reporting data about DNA damage evaluated by the alkaline Comet in pigs. Since DNA damage detected through the alkaline Comet has been shown to be linked to oxidative damage due to its relation to 8-oxo-dG, and given that DNA alterations assessed through the neutral Comet are more related to enzymatic failure (Agbaje et al., 2008; Lewis and Agbaje, 2008; Ribas-Maynou et al., 2012b; Ribas-Maynou and Benet, 2019), the use of the alkaline variant could provide additional information regarding the effects of cryopreservation, liquid-storage and antioxidants on pig sperm.

Traditionally, the evaluation of percentages of sperm with poor DNA integrity through techniques such as SCD or Comet assays has been performed in a semi-subjective manner, where the researcher categorizes sperm according to the presence/absence or halo, or the size of the Comet tail (Fernández et al., 2005; Ribas-Maynou et al., 2012a; Tandara et al., 2014). Despite the existence of defined criteria, evaluations may differ between researchers and/or laboratories. Because of that, we defined the thresholds for normal/altered DNA integrity on the basis of OTM and TD in single Comets (Table 2). ROC curves indicated that OTM was more sensitive and specific than TD for the differentiation between normal and altered cells. Therefore, this parameter was used to obtain the percentage of sperm DNA fragmentation for the previous treatments with H_2_O_2_ and DNAse (Figure 4). This percentage of altered cells showed no statistical differences between proteinase K treatments in the alkaline Comet. The explanation of this fact could reside in that we calculated and applied a threshold for each alkaline/neutral variant and every additional lysis time. Our data showed that while H_2_O_2_ led to a high percentage of altered cells determined by the alkaline Comet and a low percentage of altered cells determined by the neutral variant, the DNAse I treatment led to a high percentage of altered cells in both neutral and alkaline Comet assays (Figure 4). These data are consistent with previous reports using the Comet assay in humans (Enciso et al., 2009; Ribas-Maynou et al., 2012a) and pigs (Enciso et al., 2011).

After setting up the method, we analyzed cryopreserved samples from 11 boars. Interestingly, more incidence of DNA breaks was observed in the alkaline than in the neutral Comet (Figure 5). While no previous research using the alkaline Comet has been conducted, other studies analyzing double-strand breaks in pig sperm also found this low variability between animals (Fraser et al., 2014). In addition, the results obtained in the alkaline Comet showed a correlation with progressive motility, suggesting that the oxidative DNA break incidence could be a potential biomarker of pig sperm quality.

In conclusion, the present study demonstrates that complete chromatin decondensation is needed to evaluate the incidence of DNA breaks in pig sperm. Through setting the threshold in the OTM, measuring the percentage of sperm with fragmented DNA with the alkaline Comet may become independent from chromatin decondensation. Finally, the present work suggests that using the alkaline Comet should be explored in further studies as a sperm quality and fertility parameter.

## Data Availability Statement

The raw data supporting the conclusions of this article will be made available by the authors, without undue reservation.

## Author Contributions

JR-M conceived the study, performed the experiments, analyzed the data, performed the statistics, wrote the manuscript, revised the document, and approved the final version. AD-B and EG-B cryopreserved the samples and evaluated their sperm quality, revised the document, and approved the final version. EP critically revised the manuscript and approved the final version. MY and SB contributed to design the experiments, provided the funding, coordinated the work, revised the manuscript critically, and approved the final version. All authors contributed to the article and approved the submitted version.

## Conflict of Interest

The authors declare that the research was conducted in the absence of any commercial or financial relationships that could be construed as a potential conflict of interest.

## Footnotes

1 www.rexhoover.com

## Supplementary Material

Supplementary Figure 1 Workflow for the optimized use of the Comet assay in pig sperm.

Supplementary Figure 2 Diagram of hydrogen peroxide and DNAse I treatments conducted in this work.
