# Supplementary figures and images for: Complete Chromatin Decondensation of Pig Sperm Is Required to Analyze Sperm DNA Breaks With the Comet Assay
Source: Front Cell Dev Biol. 2021 Jun 14;9:675973. doi: 10.3389/fcell.2021.675973 (PMC8236647; doi:10.3389/fcell.2021.675973)

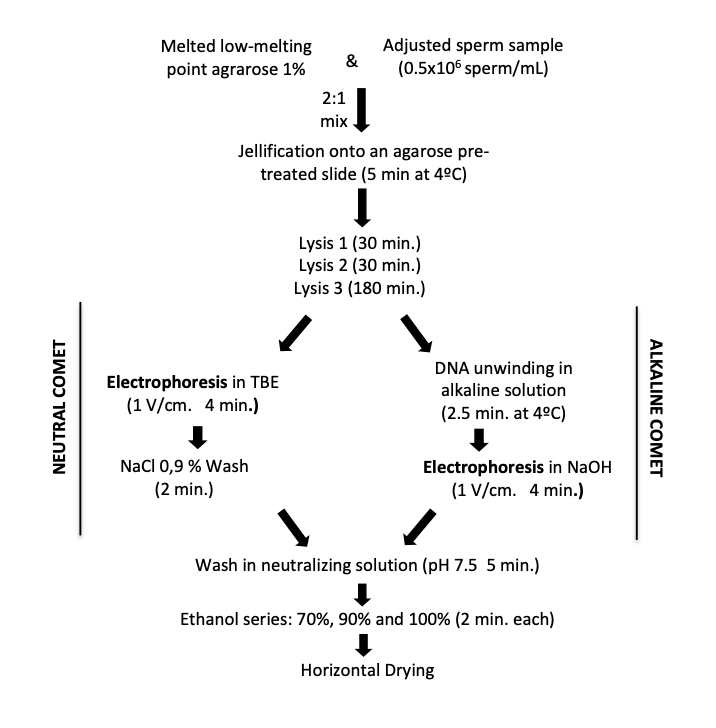

Supplement: Supplementary Figure 1 — Workflow for the optimized use of the Comet assay in pig sperm. [file Image_1.tif]

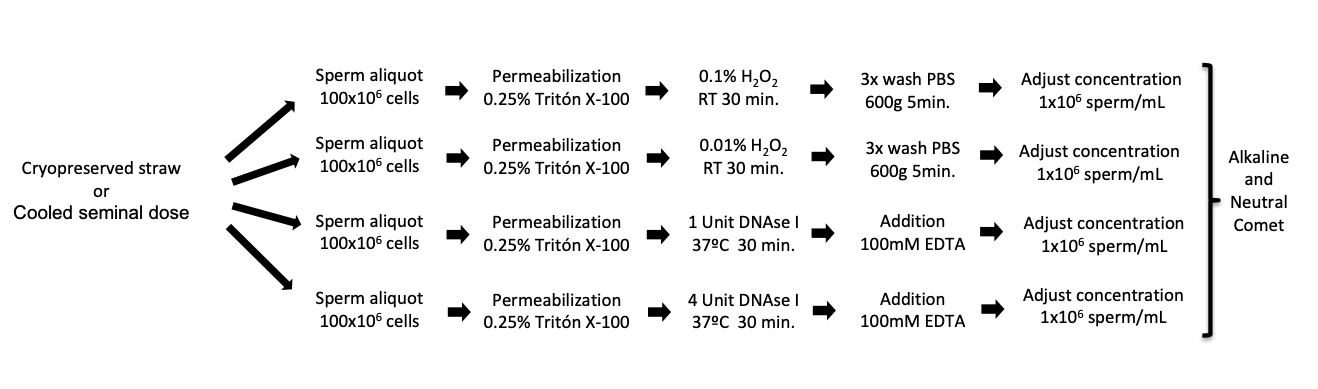

Supplement: Supplementary Figure 2 — Diagram of hydrogen peroxide and DNAse I treatments conducted in this work. [file Image_2.tif]
